# Does the merger improve the operating performance of the company? Evidence from the beverage industry in India

**DOI:** 10.12688/f1000research.139508.1

**Published:** 2023-09-11

**Authors:** Pravin Narayan Mahamuni, Shilpa Parkhi, Raju Ganesh Sunder, Kiran Karande, Samuel Gameli Gadzo, Premendra Kumar Singh

**Affiliations:** 1 Symbiosis School for Online and Digital Learning, Symbiosis International (Deemed University), Pune, Maharashtra, 412115, India; 2 Symbiosis Institute of Business Management, Symbiosis International (Deemed University), Pune, Maharashtra, 412115, India; 3 Center for Distance and Online Education, Datta Meghe Institute of Higher Education and Research, wardha, maharashtra, 442004, India; 4 Symbiosis School of Banking and Finance, Symbiosis International (Deemed University), Pune, Maharashtra, 412115, India; 5 University of Education Winneba, Winneba, Central, Ghana; 6 School of Online Education, Bharati Vidyapeeth (Deemed to be University), Pune, Maharashtra, 411030, India

**Keywords:** Merger, Acquisition, Financial Performance, Operating Performance, Beverage Industry

## Abstract

**Background:** There is fierce market competition both locally and globally. Every organisation seeks to maintain itself and, more crucially, to develop quickly through inorganic means. The expansion of a company through mergers and acquisitions is an inorganic process. Organic growth takes a very long period and is time-bound, but inorganic growth through mergers may be achieved quickly. This research aimed to determine whether the operating results of Indian beverage firms have improved after the merger or not.

**Methods:** In order to assess merger-related advantages to the acquiring firms, this study used the operating performance technique, which contrasts the pre-merger and post-merger performance of corporations using accounting data. Secondary data were used to carry out this study. The operating performance was assessed on six operating parameters (ratios) i.e. Operating Profit Margin, Gross and Net Profit Margin, Debt-Equity, Return on Net Worth and Capital Employed. The comparison was done for three years pre and post-merger period of these operating ratios.

**Results:** The findings demonstrate that mergers do not seek to increase owner wealth. This finding shows that rather than just becoming larger and achieving covert goals, managers should pay more attention to post-merger integration challenges in order to produce merger-induced synergies.

**Conclusion:** This study shows that the M&As have not had a good effect on a company’s operating performance, especially for the chosen beverage companies in India. Since financial measures cannot fully account for the influence of mergers on business performance, future research may create other metrics for merger-related gains. Research that provides profound insights into the causes and trends of post-merger business performance through the different types of mergers and industries would also be beneficial.

## Introduction

Mergers and acquisitions (M&As) in India peaked its activity levels in 2021. Many first-time buyers and an increase in industry disruptors, or insurgents, that too across multiple sectors and business activities, is what led M&As to reach such high levels ( Dezan Shira & Associates, 2022). This illustrates how the global economy is undergoing strong upheaval. In reality, this serves as a response to the changes brought about by rapid technological advancements, lower communication and transportation costs that led to the emergence of a global market, elevated competition, the emanation of new industries, a supportive financial and economic environment, and the liberalisation of the majority of economies, which too serve as motivators for mergers ( Tambi, 2005). Now a day, corporations across the globe are frequently using M&As as a business restructuring tactic. There are many studies investigating the merger phenomena, in line with the growing M&A trends ( Boateng *et al.*, 2011).

The fact that it is challenging to determine how a merger impacts the financial results of a company is a significant obstacle in completing this assignment. An alteration in profitability might have a number of causes. Mergers may produce an all-around effective reaction to a supply or demand shock in the market. They may also provide a chance to obtain cutting-edge technology or to realise economies of scale.

Even if they are significant, mergers’ impacts are still up for debate. The “market for corporate control,” which sees M&As as ways to transfer underperforming assets to companies that can use them more effectively and therefore realise the value gain, is referred to by proponents. Sceptics point out that while many mergers can be benign or advantageous, others may be driven by market dominance, arrogance, or unintentional errors, all of which have a negative impact on society. Each viewpoint is supported by evidence. The efficient-merger hypothesis appears to be supported by the regular discovery of shareholder advantages from mergers, at least in the short term, in stock market event studies. On the other hand, studies of the actual operational consequences more frequently seem to reveal that merger advantages are the exception as opposed to the rule.

### Overview of India’s Food & Beverages (F&B) industries

Globally, Indian economy is lauded as one of the quickest in terms of growth parameters. India survived the aftermath of sub-prime crisis in 2008. With a growing young and educated middle classes, which is the Indian economy’s development engine, India is predicted to surpass industrialised nations like Germany and Japan and achieve third position in the world economic rankings by the year 2030. The Indian economy underwent a dramatic structural revolution during the previous ten years as it switched from being driven by agriculture to being driven by services. Agriculture still employs 60% of the people and generates 14% of the country’s GDP. Despite the fact that the agricultural industry has advanced significantly, there are still many areas that may be improved and, if done so, would promote growth in both agribusiness and its connected industries. In order to satisfy India’s predicted significant rise in consumption over the next 10 years, agriculture and consequently the food and beverage industry would be better equipped if these challenges were addressed. With its growing economy, India’s total yearly household consumption is anticipated to quadruple, which will take India to the fifth rank by 2030 amongst the countries with the largest goods market. F&B occupy the largest space in the basket of goods consumed. This can be considered a significant accomplishment of the F&B sector in India ( Grant Thornton, 2014).

### Significance

Businesses are increasingly employing M&A (mergers and acquisitions) strategies for their regional and worldwide development in order to expand their company scope or seize new possibilities ( Ferreira *et al.*, 2016). Given the context, this research effort has been made to investigate, observe, and evaluate the operational results of the Indian beverages sector with regard to United Spirits Limited and United Breweries Limited, which have participated in M&A activities following the post liberalization, privatization, globalisation (LPG) era in India, and to ascertain whether M&As significantly affected the financial operating performance of merging entities. The purpose of this study is to investigate M&A in the beverage sector in India to analyze whether there were differences in outcomes for various companies operating within the same sector.

## Literature review

Our study assesses the consequences of mergers on competition. Various studies have found varying effects from mergers in various industries, which is not surprising. A variety of research has been conducted about the association between M&As and business performances ( Bi Z., 2016). Using several types of financial (such as profits and stock prices) and non-financial (such as the reputations of the firms) indicators and of course, the time periods (such as initial market reaction to the M&As, pre and post-measurement, etc.). According to these studies, M&A deals often benefit the target’s shareholders more than the acquirer’s shareholders. In reality, the performance of the buying firm generated a variety of outcomes ( Schweiger and Very, 2003).

The 50 biggest mergers in the US between 1979 and 1984 were quantified and their cash flow performance was assessed by Healy, Palepu, and Ruback in 1992. They found that, compared to their respective industries, *the operating performance of merging companies substantially enhanced in post-merger period* ( Healy *et al.*, 1992).

In 1983, Katsuhiko Ikeda *et al.* examined the financial results of forty-three (43) combining enterprises from the manufacturing sector in Japan. In more than half of the cases, they noticed an increased Return on Equity (RoE), whereas only approximately half the cases saw an improvement in the rate of return on total assets. However, “ *both profit rates improved in more than half of the cases in the five-year test, indicating that improvements in firm performance after mergers began in line with internal adjustments made by the merging firms. This suggests that there was a necessary gestation period during which merging firms learned how to manage their new businesses”* ( Ikeda and Doi, 1983).

The impact of M&As on the financial health of 40 United Kingdom corporations were researched by Jallow, Masazing, and Basit (2017) between the years of 2006 and 2010. According to the analysis, M&As had “ *a large influence on ROA, ROE, and EPS but a negligible impact on NPM”.* The study concluded that a lack of managerial effectiveness, an inefficient utilisation of shareholders’ funds, and escalated financial costs are responsible for companies’ insignificant decreases in Return on Assets (RoAs) and RoEs after the mergers took place ( Jallow *et al.*, 2017).

Between 1995 and 2000, Beena (2000) used a set of financial ratios 4 and a t-test to compare the performances of a sample of 115 acquirers, from Indian industrial sector, before and after the merger. “ *The investigation was unable to identify any proof that the financial ratios for the acquiring corporations had improved in the post-merger era compared to the pre-merger period”* ( Beena, 2000).

The financial holding companies’ post-merger banks generated merger synergies. The top 10 banks in financial holding companies and top 10 banks in non-financial holding companies revealed that 3 out of the top 10 financial holding company banks were founded in the banking industry and are connected to financial holding companies that place a strong emphasis on banking. This finding indicates that financial holding companies perform better overall, post-merger, if banking is their primary operating entity ( Liu, 2010).

The study examines a few financial parameters (ratios) before and after a merger of Indian F&B industry acquiring firms to determine the effects of M&As on their operating financial results. The outcome refers to a minor, but not statistically significant, *improvement in profitability ratios in the food industry.* While the return on invested capital and net worth have decreased. In the post-merger period, both the food and beverage industries have seen a negligible hike in leverage. Post-merger, the combined *performance of the food and beverage companies improved significantly,* but statistical analysis cannot determine whether the mean of the two variables differed significantly ( Mahamuni and Jumle, 2012).

Mahamuni and Jumle, in 2018, carried out a study of manufacturing machinery and metal products firms to verify if M&A activity helps the firms in improving their performances after the merger in terms of parameters like improvements in liquidity position, better solvency scenarios, expansion of their businesses, overall improvement in profitability. The result revealed that manufacturing companies which merged “ *did not achieve liquidity, solvency, profitability after merger”.* Also, it is seen that *after the merger, the operating results of the combined manufacturing firms has not improved.* But the merged companies, post-merger, expanded their business activities ( Mahamuni and Jumle, 2018).

According to research carried out by Pramod Mantravadi and A Vidyadhar Reddy (2008), mergers appear to have experienced “ *a marginally positive impact on the profitability of businesses in the banking and finance sector, while they had a marginally negative impact on operating performance (in terms of profitability and returns on investment) for businesses in the pharmaceutical, textile, and electrical equipment sectors”.* In terms of profitability margins, ROI, and asset values, the Chemicals and Agri-products industries had experienced a significant decrease due to mergers ( Mantravadi and Reddy, 2008).

The study by Ahmad Ismail, Ian Davidson, and Regina Frank (2009), is focused on European banks. It examined *operating performance following the merger event*, and found that the industry-adjusted average cash flow return was not substantially changed after the merger but remained positive. Additionally, it was observed, low profitability, conservative credit policies, and robust cost-efficiency status in pre-merger period, which provided the source for increasing these returns post-merger are the major predictors of industry-adjusted cash flow returns ( Ismail *et al.*, 2009).

Mahesh Kumar Tambi (2005) took forty companies’ database from *CMIE’s PROWESS and applied a paired t-test* for mean differences for 4 parameters viz. total performance improvement, economies of scale, operating synergy and financial synergy. He investigated the impact of mergers on Indian enterprises. *The investigation indicates that Indian companies are comparable to those in various places of the globe and that mergers did not significantly increase performance* ( Tambi, 2005).

Using measures 5 of profitability, growth, leverage, and liquidity, Pawaskar V. (2001) focused on the before and after the merger operating performance of 36 acquiring firms between 1992–1995 and revealed that these *firms surpassed the profitability average for the sector.* Regression analysis, though, discovered that *growth in the profitability did not shown growth following the merger period when compared to the acquiring firms’ top rivals* ( Pawaskar, 2001).

Sinha, Kaushik and Timcy (2010) conducted the study to measure Post Merger and Acquisition Performance. Through this research, they investigated selected organizations from Financial Sector in India. With an aim to understand how M&As sway the financial performance of the select Indian ‘Financial Institutions’. The researchers discovered that M&As incidents in India showed “ *a significant correlation between financial performance and the M&A deal”*, in the long-run, along with the fact that the acquiring firms could generate value ( Sinha *et al.*, 2010).

According to a 2009 study by Murugesan, Manivannan, Gunasekaran, and Bennet titled “Impact of Mergers on the Corporate Performance of Acquirer and Target Companies in India,” *the acquirer businesses’ shareholders improved their liquidity performance following the merger event* ( Selvam *et al.*, 2009).

Marina Martynova, Sjoerd Oosting and Luc Renneboog (2006) looked at the long-term profitability of business takeovers in Europe and observed that *“both acquiring and target companies significantly outperformed the median peers in their industry prior to the takeovers, but the profitability of the combined firm decreased significantly following the takeover”* ( Marina *et al.*, 2006).

From 1993 to 2010, Sinha and Gupta (2011) examined the effects of M&As on the Indian financial sector. 80 companies that went through M&A over the past 18 years were examined in the study. The reveals that M&As had ‘ *a favorable impact on profitability’* represented by the net profit and the ratio of profit before interest, tax, depreciation and amortization (PBITDA), ‘ *a negative effect on liquidity’*, also decreased total and systematic risk ( Sinha and Gupta, 2011).

Abdullah Mamun, George Tannous, Sicong Zhang (2021) studied how bank mergers (regulatory mergers) performed (operating) post-merger during and after the 2008-2009 financial crisis. Up to two years after the acquisition, regulated mergers are *seen to significantly increase profitability and cost effectiveness.* In comparison with rivals who were not involved in the merger, these improvements are significantly higher. However, the operating result of non-regulatory mergers following the merger does not differ substantially from that of their non-merger peers ( Mamun *et al.*, 2021).

The impact of mergers when businesses compete on pricing and cost-cutting efforts was examined by Motta and Tarantino (2021). They discover that following the merger, overall investments and consumer surpluses are lower when efficiency benefits are missing. Only when efficiency improvements are substantial enough are the impacts of a merger competitively advantageous. The effect of horizontal mergers that lead to monopolies on businesses’ incentives to engage in demand-enhancing innovation is examined to discover that a merger’s overall effect on innovation might be either favourable or unfavourable ( Motta and Tarantino, 2021).

Numerous research papers have been examined, and it has been determined that the impacts on financial results are inconsistent, mixed, and different depending on the industry. The fact that the researchers’ methodologies varied made it difficult to summarise their findings, as in some of their results, they used a variety of variables, parameters, and financial information. Financial performance metrics were employed in several studies. The majority of studies that employed financial performance measures found no appreciable differences (on either side) between the financial performance prior to and following the M&A.

These preceding works of research might be used to draw the conclusion that mergers generally do not appear to enhance the post-merger efficiency of acquirers. Gains are either negligible or non-existent by various measurements. Event studies and accounting both fail to provide any proof of value generation. This study’s goal is to investigate these theories in the context of India. There are few studies on the performance following a merger of Indian corporations and consequently a large knowledge gap in this field. The operating efficiency technique is utilised in this study to determine how a merger will affect the efficiency of acquiring organisations.

## Research objectives

Based on the literature review, the researcher frames the objective and the hypothesis to carry-out the study as below;

**Objective:** To measure, compare and study the merger’s impact on the operating performance.

**Hypothesis:** Merged firms have improved their operating performances.

## Methods

According to several merger studies, evaluating and comparing the merged firms to a similar industry group that is based on the performance before and after the merger is an effective way to find operating performance improvements ( Behr & Heid, 2011; Fee & Thomas, 2004; Ghosh, 2001; Powell & Stark, 2005). The operating performance is assessed on six operating parameter (ratios) i.e. Operating Profit Margin, Gross and Net Profit Margin, Debt-Equity, Return on Net Worth and Capital Employed. The required financial data are extracted through the Centre for Monitoring Indian Economy (CMIE) Prowess Database. The comparison of three years pre and post-merger period of these operating ratios ( Aggarwal and Garg, 2022).

### Research design

The researcher used an analytical and quantitative research design to measure and compare the operating result before and after the merger period.

### Data source

All data used in this paper can be found at the Centre for Monitoring Indian Economy (CMIE) Prowess Database (Version Prowess IQ v3.0). The relevant data of three years period pre-merger and post-merger, considering the merger year as the baseline year i.e. 0 (zero) were used.

Research papers, reports of research organization and books used for the study are mentioned in references with URLs.

### Sampling method

The researcher followed the non-probability convenient sampling method to select firms from the Indian beverage industries. United Spirits Limited (USL) and United Breweries Limited (UBL) were selected for the study for the reason that they are two of the most renowned and prestigious companies in India’s beverage industry.

### Data analysis and tools used

For all the sample firms that underwent mergers, operating performance ratios both before and after the merger were estimated, and averages (mean) were computed and compared in order to assess the merger’s impact and using a “paired two sample t-test” with a confidence level of 0.05, it was determined whether there had been any statistically significant change in operating performance as a result of mergers. Mean, paired ‘t’-test, and Ratio Analysis, are a few of the methods applied for analyzing and assessing the data that was collected. SPSS (Statistical Package for the Social Sciences), also known as IBM SPSS Statistics. IBM SPSS Statistics Base 29.0 was used for data processing and paired ‘t’ test analysis.

## Results and discussions

Study of historical cases of Mergers and Acquisitions have established the changes in financial performance of the restructured firms. However, the previous studies and the current paper confirm that the firm’s performance in terms of profitability, liquidity, and solvency does not show any significant improvement in the short run in the post-merger period. The present study of post-merger activity’s long-term effects may add interesting results which can yield a future research dimension.

### Operating profit margin

Table 1 shows that the Operating Profit Margin (the mean) of both beverage companies is much lower after the merger than it was before the merger. However, in the instance of United Breweries Limited, the operating profit margin post-merger is statistically worse (-14.88 in the pre period and -24.55 in the post period, t-value = 4.964, ‘p’= 0.05). This means both companies are unable to control their costs, and as a result, operational profit after the merger is lower. After covering all operational expenses, operating profit will typically decline throughout the post-merger period.

**Table 1.** Operating Profit Margin.

Company		UBL	USL
**Pre-merger**	**-3**	-16.47	-31.26
	**-2**	-14.86	-38.51
	**-1**	-13.32	-30.27
**Post-merger**	**1**	-23.36	-39.53
	**2**	-23.55	-39.51
	**3**	-26.74	-44.75
**MEAN**	**Pre**	-14.88	-33.35
	**Post**	-24.55	-41.26

### Gross profit margin

Gross Profit Margin from Table 2 indicates that United Breweries Limited’s mean (pre-merger: 8.38; post-merger: 6.48) is down, while United Spirits Limited’s mean (pre-merger: 1.61; post-merger: 0.69) is up, indicating that both companies can recover their costs from sales (COGS). However, since the ‘p’ value is more than 0.05, changes in the gross profit margins of the two companies are statistically insignificant.

**Table 2.** Gross Profit Margin.

Company		UBL	USL
**Pre-merger**	**-3**	6.74	3.45
	**-2**	9.13	4.62
	**-1**	9.28	14.61
**Post-merger**	**1**	6.69	8.43
	**2**	6.23	8.85
	**3**	6.53	7.75
**MEAN**	**Pre**	8.38	7.56
	**Post**	6.48	8.34

### Net profit margin

In Table 3, the Net Profit Margin of acquired businesses during the pre-merger and post-merger periods is shown. The average Net Profit Margin ratio for United Breweries Limited (1.55 in the pre and 2.93 in the post-period) is improving, indicating that the company is better at converting sales to actual profit, but at the required probability level, the gain is not statistically significant. The Net Profit Margin (the mean) of United Spirits Limited, on the other hand, has dropped in after the merger; at the required probability level, the decline is not significant, indicating that the net profit margin has reduced, rather than increased, post-merger. It indicates that not all of the activities are carried out efficiently.

**Table 3.** Net Profit Margin.

Company		UBL	USL
**Pre-merger**	**-3**	0.48	1.45
	**-2**	2.08	1.15
	**-1**	2.1	9.83
**Post-merger**	**1**	3.11	3.94
	**2**	2.49	4.04
	**3**	3.2	2.97
**MEAN**	**Pre**	1.55	4.14
	**Post**	2.93	3.65

### Return on net worth

Table 4 provides the sample merged firms’ average Return on Net worth over the pre and post-merger periods. It should be noticed that the variation in average returns on net worth for both of the chosen merged corporations, i.e. United Breweries Limited (pre-merger =19.47 and post-merger = 13.34), and United Breweries Limited (pre-merger = 20.38 and post-merger = 9.67), is lower after following merger as compared to before the merger event Therefore, it may be concluded that although net value has increased substantially as a result of M&As, the merged companies were incapable of delivering the necessary returns on their net worth post-merger.

**Table 4.** Return on Net Worth.

Company		UBL	USL
**Pre-merger**	**-3**	7.92	9.55
	**-2**	37.49	7.15
	**-1**	13	44.45
**Post-merger**	**1**	18.51	11.66
	**2**	10.42	9.56
	**3**	11.1	7.8
**MEAN**	**Pre**	19.47	20.38
	**Post**	13.34	9.67

### Return on capital employed

The average value of return on capital employed by United Spirits Limited has gone down from 8.36 (before merger) to 5.80 (after merger), based on the analysis of Table 5 of both the Sample Merged Firms during the before and after Merger Periods. Nevertheless, in the case of United Breweries Limited, return on capital employed has improved from 2.26 (before merger) to 5.21 (after merger). It suggests this company, following the merger, proved efficient in utilising its funds. Additionally, it indicates that management exhibited efficiency in employing investments and the creditors. Furthermore, the derived ‘t’ values for the above two firms, at the required degree of probability, are not statistically significant, nor is an increase or decrease in the ratio.

**Table 5.** Return on Capital Employed.

Company		UBL	USL
**Pre-merger**	**-3**	0.88	3.67
	**-2**	2.9	2.55
	**-1**	3	18.86
**Post-merger**	**1**	5.73	7.24
	**2**	4.39	5.64
	**3**	5.5	4.53
**MEAN**	**Pre**	2.26	8.36
	**Post**	5.21	5.80

### Debt equity ratio

The findings from the analysis of Table 6 of the sample merged firms’ debt-equity ratios for the pre- and post-merger periods show that United Breweries Limited’s debt-equity ratio was substantially reduced from 1.61 (before merger) to 0.69 (after merger), t-value = 3.267 and p > 0.05). It clarifies that a large portion of assets after the merger are financed by debt rather than equity. It indicates that these companies are embarking on more debt as a result of merger activity.

**Table 6.** Debt to Equity Ratio.

Company		UBL	USL
**Pre-merger**	**-3**	10.98	2
	**-2**	12.87	1.73
	**-1**	1.92	1.11
**Post-merger**	**1**	2.15	0.63
	**2**	1.03	0.73
	**3**	1.01	0.71
**MEAN**	**Pre**	8.59	1.61
	**Post**	1.40	0.69

## Hypotheses testing

The hypothesis that the Operating performance of the merged firms has improved has been rejected after examining the results mentioned above. As Table 7 clearly indicates, the sample companies’ operating performance has declined as a result of the merger activity they undertook. It reveals a negative impact on the sample companies’ overall profitability over the post-merger period.

**Table 7.** Overview of the Performance Parameters.

Performance Parameters	UBL	USL
Operating Profit Margin	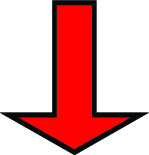	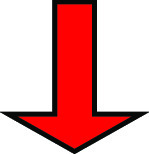
Gross Profit Margin	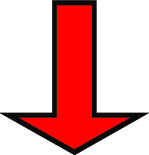	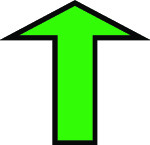
Net Profit Margin	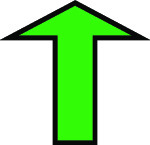	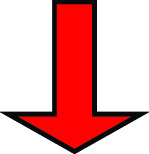
Return on Net Worth	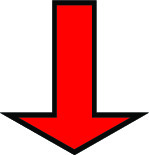	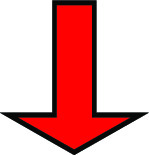
Return on Capital Employed	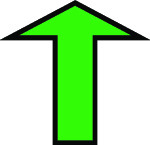	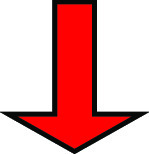
Debt – Equity Ratio	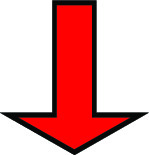	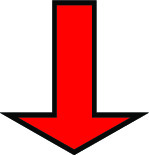

The representative sample firms from the beverage industry’s post-merger operating performance is declining. Profitability ratios are declining along with general falloffs in returns on net worth and capital invested. The earnings ratios for United Breweries Limited have somewhat improved, albeit not statistically significantly. For United Spirits Limited, the return on net worth and investment made has declined. A negligible increase can be observed in leverages of both of the firms that belong to the Indian beverage sector that were picked during the period following the merger. On the reverse hand, this ratio of debt to equity has decreased dramatically since the merger compared to prior. It suggests that debt rather than equity is used to fund a large part of assets in the post-merger era. It demonstrates that these businesses are increasing their debt loads as a result of merger activity. Overall, it can be said that there was a negative on operating performance by the merger activity done by these two representative sample businesses in India’s beverage industry.

One may draw the conclusion that the three financial variables included in this study do not statistically significantly vary the operating results after merging. The null hypothesis is true because all estimated t-values are lower than (or more on the negative side of) the table value. This outcome indicates that merger activities do not affect the acquired businesses’ operational performance.

Another reason why profitability did not increase after a merger is because acquisition of a company could have led to “managerial control loss problems.” One may argue that the acquirers encounter unforeseen difficulties while handling and integrating their purchases. The acquiring leadership loses control and is unable to manage the merged firm effectively as it grows more complex. After a merger, profitability levels fall as a result of this loss of control.

## Conclusion

This research work was conducted to better understand the impact of M&As activity on operating financial results. This study shows that the M&As have not had a good effect on a company’s operating performance, especially for the chosen beverage companies in India. Despite the limited favourable effects, they are statistically negligible.

Although there are many motivations for a firm to participate in merger activity, our aim in this search was to understand one crucial feature of M&A activity. It might be difficult to interpret the conclusion or get insights from the quantitative information when these motives or reasons are qualitative. Additionally, it has been noted from several studies that merger and acquisition efforts for numerous organisations in their post-merger phase did not result in beneficial short-term effects. However, if the companies do not conduct thorough research before deciding on M&As, it will not meet their expectations or the objectives for the activity.

By calculating it and comparing it to the average for an industry or sector, subsequent studies in this area may expand on the present study. Any variations, if any, may then be further investigated to get a better understanding. The results of research demonstrating inferior performance in the post-merger era might be compared and connected to post-merger return to investors of acquiring corporations who are part of mergers taking place in India.

Previous studies have shown that there was no significant improvement in business performance. It was discovered that merger-induced changes in a company’s industry-adjusted profitability, asset efficiency, and solvency status were statistically negligible. This finding suggests that mergers do not increase the acquirers’ operating performance. These empirical findings allow us to draw the conclusion that merger decisions are not made with the intention of maximising shareholder value through increased profitability. The pursuit of larger scale, market consolidation, and empire building may have served as inspirations for merger choices.

There may occasionally be unstated goals, such as the post-merger asset stripping of the target firm that provides the promoters with a significant cash premium over and above the net worth.

In order to accomplish the true goals of the merger, management must continue to concentrate on the company’s operations, especially the post-merger integration phase.

Industry mergers and acquisitions do not appear to be slowing down. Why do businesses choose mergers and acquisitions (M&A) when, on average, data shows that doing so would hurt them more than help the target? It appears to point to some issues with the conceptual framework, the technique, or the accuracy of the data. This topic may be explored in more detail.

Financial metrics may not fully reflect the impact of mergers on business performance or reveal the driving forces behind M&A decisions. Therefore, in future research, the post-merger performance gains might be examined in terms of some additional criteria including social value provided, improvements in gains to other stakeholders of the firms engaging in M&A, and advantages at the industry and economy level at both the national and worldwide levels.

### Limitations of the study

Due to several limitations, the research only offers a few explanations for why there was no merger-induced increase in corporate performance. Furthermore, the article does not examine the results to see if any trends in post-merger performance across merger types and industries exist. Future research might address concerns in these areas.

## Data Availability

Data used in this study are from the CMIE ( *Centre for Monitoring Indian Economy* Pvt. Ltd. India). The datasets of the Indian Companies are available from the Centre for Monitoring Indian Economy (CMIE) Prowess Database (Version Prowess IQ v3.0). https://prowessiq.cmie.com/. Anybody who wishes to use the data can register and use the data for academic purposes. All data were collated through annual reports of Companies. A guide for how to apply for dataset access is available at: https://register.cmie.com//kommon/bin/sr.php?kall=wcontactus&tab=2060&rrurl=prowessiq.cmie.com. The data we extracted for the study are as follows:
