# Supplementary material for: Does the merger improve the operating performance of the company? Evidence from the beverage industry in India
Source: F1000Res. 2023 Sep 11;12:1130. [Version 1] doi: 10.12688/f1000research.139508.1 (PMC10837612; doi:10.12688/f1000research.139508.1)
Supplement: Peer review history

# Reviewer report: Reviewer response for version 1

**DOI:** 10.5256/f1000research.152785.r208030

**Published:** 2023-11-06

**Authors:** H M Belal

**Affiliations:** 1 School of Business & Management, Liverpool Business School, Liverpool John Moores University, Liverpool, England, UK

The application of a Merger and Acquisition (M&A) strategy serves as a substantial and consequential method for maintaining business resilience in highly competitive market environments. Nonetheless, the challenge of assessing a company's performance through the lens of M&A strategies is a complex and multifaceted task. This research endeavor has been undertaken to substantiate the rationale behind the utilization of M&A strategies in the Indian beverage industry. Nevertheless, it is important to acknowledge the forthcoming constructive observations and recommendations designed to elevate the overall quality of the research paper.

 Introduction:

1. The rationale behind the research issues is presently insufficiently established. To enhance this aspect, it is recommended to augment the reference base with a more extensive array of scholarly sources. Simultaneously, it is advisable to place increased emphasis on both elaborating upon these concerns and providing a comprehensive contextual backdrop for the study.
2. I did not find any research objectives in the Introduction part. Please develop the Research objectives based on the Research problem and Research questions.
3. So, the introduction section requires a redesign, and there is a need for improvement in the academic writing style.

Literature Review:

1. In the literature review, the authors are encouraged to construct compelling discussions on the research objectives, drawing upon the insights of esteemed scholars. It is vital to ensure this practice is consistently followed.
2. There are lack of using range of relevant literature. Critical evaluation of key concepts and theories is also required.
3. Research Objectives should be included in the Introduction part and the Research questions.

Research Methodology:

1. The research design lacks adequate information, and it is essential that a more comprehensive explanation is provided.
2. The description of the sampling method is deficient in detail, necessitating a thorough exposition of the rationale for opting to utilize CMIE. Clarification in this regard is indispensable.

Discussion:

1. Kindly proceed with the formulation of the Discussion section, utilising the results and findings as the basis.
2. In Discussion, authors need to justify the research results through scholars' references.

Is the work clearly and accurately presented and does it cite the current literature?

Partly

If applicable, is the statistical analysis and its interpretation appropriate?

Yes

Are all the source data underlying the results available to ensure full reproducibility?

No source data required

Is the study design appropriate and is the work technically sound?

Partly

Are the conclusions drawn adequately supported by the results?

Partly

Are sufficient details of methods and analysis provided to allow replication by others?

Partly

Reviewer Expertise:

Operations Management

I confirm that I have read this submission and believe that I have an appropriate level of expertise to confirm that it is of an acceptable scientific standard, however I have significant reservations, as outlined above.

# Response

**Published:** 2023-11-16

**Authors:** Raju Ganesh Sunder

**Affiliations:** FoCMS, Datta Meghe Institute of Higher Education and Research (Deemed University), Wardha, Wardha, Maharashtra, India

I did not find any research objectives in the Introduction part. Please develop the Research

 objectives based on the Research problem and Research questions. – Purpose is mentioned in the

 Significance in the sub-section of the Introduction.

 In the literature review, the authors are encouraged to construct compelling discussions on the

 research objectives, drawing upon the insights of esteemed scholars. It is vital to ensure this practice

 is consistently followed – Discussion and summary of the literature review is mentioned in the last 3

 paragraphs of the Literature.

 Research Objectives should be included in the Introduction part and the Research questions. –

 Mentioned after the Literature Review

 A thorough exposition of the rationale for opting to utilize CMIE – Updated in data source

 In Discussion, authors need to justify the research results through scholars' references - Added in

 Results and Discussion section

# Reviewer report: Reviewer response for version 1

**DOI:** 10.5256/f1000research.152785.r208032

**Published:** 2023-10-13

**Authors:** Anurag Bhadur Singh

**Affiliations:** 1 School of Commerce, XIM University, Bhubaneswar, India

**Followings are the observation, authors may incorporate to improve the quality of papers:**

**Research objectives**: Objectives can be separated for comparison and measure purpose.

**Hypothesis: **can also develop separate as objectives describes.

**Research Methodology:**

1. More details may be given in this section such as no. of firms, no of years of study i.e. from 2005-2015.
2. Should justify why only two firms has selected for the study, justify the same with citations.

**Data Analysis:**

1. Authors should justify, why only paired two sample t-test opted for analysis purpose, with some citations.
2. Table 1, 2, 3, 4, 5, should contains S.D., N, also because to apply t test we need all these information.
3. A separate table can be develop for t test containing degree of freedom and t stat. value. why it test only at 5% level of significance only with some citations.
4. Debt equity table not required in the study because of role are different of this ratio.
5. Trend analysis can also be useful for measure purpose, authors may use this in this section.
6. From page no. 11 seems that authors has selected only three years for comparison of performance of the firms which seems unjustified, must be cite some study who did the same.
7. My suggestions that authors may use at least five years each pre and post M&A data to set the comparison in performance, because in short periods of time comparison can not be done specially M&A has taken place or corporate restructuring has done by the entity.

**Conclusion:**

1. Authors should cite some study who has concluded the same results. or may justify why this study results are different from them.

If all above suggestions will incorporate in the study, i am sure the quality of paper will improve.

 With Best wishes.

Is the work clearly and accurately presented and does it cite the current literature?

Yes

If applicable, is the statistical analysis and its interpretation appropriate?

Partly

Are all the source data underlying the results available to ensure full reproducibility?

No

Is the study design appropriate and is the work technically sound?

Partly

Are the conclusions drawn adequately supported by the results?

Partly

Are sufficient details of methods and analysis provided to allow replication by others?

Partly

Reviewer Expertise:

Corporate Finance, Accounting, Economic developments

I confirm that I have read this submission and believe that I have an appropriate level of expertise to confirm that it is of an acceptable scientific standard, however I have significant reservations, as outlined above.

# Response

**Published:** 2023-11-16

**Authors:** Raju Ganesh Sunder

**Affiliations:** FoCMS, Datta Meghe Institute of Higher Education and Research (Deemed University), Wardha, Wardha, Maharashtra, India

Objectives can be separated for comparison and measure purpose - Thank you for this point, now it

 is done

 More details may be given in this section such as no. of firms, no of years of study i.e. from 2005-

 2015 - Thank you for suggestions, we have added in data section

 Should justify why only two firms has selected for the study, justify the same with citations - Thank

 you for the point mentioned, we have added in Sampling section

 Authors should justify, why only paired two sample t-test opted for analysis purpose, with some

 citations - Thank you now we have added in Data Analysis section

 Table 1, 2, 3, 4, 5, should contains S.D., N, also because to apply t test we need all these information

 - Thank you we have added SD in the respective table

 From page no. 11 seems that authors has selected only three years for comparison of performance

 of the firms which seems unjustified, must be cite some study who did the same - Thank you, we

 have added in Limitations section

 My suggestions that authors may use at least five years each pre and post M&A data to set the

 comparison in performance, because in short periods of time comparison cannot be done specially

 M&A has taken place or corporate restructuring has done by the entity - Thank you we have added

 in Limitations section

 Authors should cite some study who has concluded the same results. or may justify why this study

 results are different from them - Thank you we have added in Results and Discussion section

# Reviewer report: Reviewer response for version 1

**DOI:** 10.5256/f1000research.152785.r208033

**Published:** 2023-10-04

**Authors:** Priyanka Tandon

**Affiliations:** 1 Regenesys Business School, Sandton, South Africa

**Brief description of an article**

 This study employed the operating performance technique, which compares the pre-merger and post-merger performance of organizations using accounting data, to determine the benefits of mergers to the acquiring company. Using secondary data six operating metrics (ratios), namely Operating Profit Margin, Gross and Net Profit Margin, Debt-Equity, Return on Net Worth, and Capital Employed, were used to evaluate the operating performance. These operating ratios were compared for three years before and after the merger.

**Relevance of an article**

*Major concerns*

1. In section-1, author(s) failed to address the research objectives and questions pertaining to the research study. The research objectives which is actually mentioned after LR should be the sub-part of section-1.
2. Ideally, the introduction section must clarify the gaps, the objectives, the guiding research questions, and the philosophical stance. In the current form, the introduction section fails to meet any one of these criteria. Moreover, the authors’ research questions do not conform to the study.
3. Author(s) must discuss at least three contributions of study (theoretical and practical) which must be convincing towards the novelty of the study.
4. Author(s) must mention the structure/organization of study.
5. The literature review is quite descriptive. It lacks critical debates that help shape the theoretical debates which are essential for scientific arguments. I am sorry but the literature review is prepared like a thesis chapter. Moreover, research gaps, research questions, and the research objectives should be presented in the introduction section.
6. Discussion should be separate section after results and must specifically discuss the results (linkage to similar and contrary studies) and also implications of study in detail.

*Minor concerns*

 I found typos and inconsistencies in citations which should be carefully sorted out.

 Overall, the manuscript is good and it can be indexed after incorporating the above suggestions.

Is the work clearly and accurately presented and does it cite the current literature?

Yes

If applicable, is the statistical analysis and its interpretation appropriate?

Yes

Are all the source data underlying the results available to ensure full reproducibility?

Yes

Is the study design appropriate and is the work technically sound?

Yes

Are the conclusions drawn adequately supported by the results?

Yes

Are sufficient details of methods and analysis provided to allow replication by others?

Yes

Reviewer Expertise:

Finance

I confirm that I have read this submission and believe that I have an appropriate level of expertise to confirm that it is of an acceptable scientific standard, however I have significant reservations, as outlined above.

# Response

**Published:** 2023-11-16

**Authors:** Raju Ganesh Sunder

**Affiliations:** FoCMS, Datta Meghe Institute of Higher Education and Research (Deemed University), Wardha, Wardha, Maharashtra, India

In section-1, author(s) failed to address the research objectives and questions pertaining to the

 research study - Thank you for the point, we have added in Significance section of Introduction

 Ideally, the introduction section must clarify the gaps, the objectives, the guiding research questions,

 and the philosophical stance - Thank you for the suggestion we have added in Research Question

 section

 Author(s) must discuss at least three contributions of study (theoretical and practical) - Thank you

 for the point we have added in contribution section after the limitation of the study

 Author(s) must mention the structure/organization of study - Thank you now, added at end of

 Introduction section

 The literature review is quite descriptive. Moreover, research gaps, research questions, and the

 research objectives should be presented in the introduction section - We thank you for the

 suggestion, both Literature Review and Introduction section revised and updated

 Discussion should be separate section after results and must specifically discuss the results and also

 implications of study in detail - This is done

 I found typos and inconsistencies in citations - Thank you for this now it is corrected
